# Analysis of the mechanism of nucleosome survival during transcription

**DOI:** 10.1093/nar/gkt1120

**Published:** 2013-11-13

**Authors:** Han-Wen Chang, Olga I. Kulaeva, Alexey K. Shaytan, Mikhail Kibanov, Konstantin Kuznedelov, Konstantin V. Severinov, Mikhail P. Kirpichnikov, David J. Clark, Vasily M. Studitsky

**Affiliations:** ^1^Department of Biochemistry and Molecular Biology, UMDNJ-Robert Wood Johnson Medical School, 675 Hoes Lane, Piscataway, NJ 08854, USA, ^2^School of Biology, Lomonosov Moscow State University, 119991 Leninskie gori, MSU, Bldg. 1, korpus 12, Moscow, Russia, ^3^Waksman Institute of Microbiology, Rutgers, the State University of New Jersey, 190 Frelinghuysen Road, Piscataway, NJ 08854, USA, ^4^Institute of Gene Biology, Russian Academy of Sciences, 1190334 34/5 Vavilova street, Moscow, Russia and ^5^Program in Genomics of Differentiation, National Institute of Child Health and Human Development, National Institutes of Health, PO Box 3006, Rockville, MD 20847, USA

## Abstract

Maintenance of nucleosomal structure in the cell nuclei is essential for cell viability, regulation of gene expression and normal aging. Our previous data identified a key intermediate (a small intranucleosomal DNA loop, Ø-loop) that is likely required for nucleosome survival during transcription by RNA polymerase II (Pol II) through chromatin, and suggested that strong nucleosomal pausing guarantees efficient nucleosome survival. To evaluate these predictions, we analysed transcription through a nucleosome by different, structurally related RNA polymerases and mutant yeast Pol II having different histone-interacting surfaces that presumably stabilize the Ø-loop. The height of the nucleosomal barrier to transcription and efficiency of nucleosome survival correlate with the net negative charges of the histone-interacting surfaces. Molecular modeling and analysis of Pol II-nucleosome intermediates by DNase I footprinting suggest that efficient Ø-loop formation and nucleosome survival are mediated by electrostatic interactions between the largest subunit of Pol II and core histones.

## INTRODUCTION

Passage of RNA polymerase II (Pol II) is accompanied by efficient recovery of nucleosomal structure—a process that is essential for proper gene regulation, cell survival (1,2) and normal aging (3). During moderate-level Pol II transcription *in vivo* histones H2A/H2B are constitutively displaced/exchanged whereas histones H3/H4 are displaced/exchanged only during intense transcription [(4–13) and see (14,15) for review].

Nucleosome recovery during moderate-level Pol II transcription has been recapitulated *in vitro*: during nucleosome traversal a single H2A/H2B dimer is released, but the remaining subnucleosome (hexasome, DNA-bound histone hexamer) survives after transcription (16–19). We have proposed that formation of the Ø-loop-containing intermediate at the position +49 is essential for efficient nucleosome survival during Pol II transcription (20). Furthermore, it has been proposed that electrostatic Pol II–histone interactions could stabilize the Ø-loop intermediate and thus facilitate nucleosome survival during transcription (20).

To evaluate this possibility, in the present work we classified various RNA polymerases (RNAPs) that are structurally similar to Pol II according to net charge and sequence conservation of the predicted histone-interacting region (zero-loop-stabilizing sequence: ZLS sequence). Next, we analysed the transcription efficiencies, nucleosome survival and structures of the elongation complexes formed at the position +49 (EC+49) by these enzymes. The sequence conservation and net negative charges of the ZLS sequences positively correlated to the efficiency of nucleosome survival, the intensity of +45 pausing and the formation of the Ø-loop intermediate at the position +49. We have also examined the effects of mutations on the ZLS region of yeast Pol II during transcription through a nucleosome. The data suggest that electrostatic interactions between Pol II and histones stabilize the Ø-loop intermediate during transcription and promote nucleosome survival.

## MATERIALS AND METHODS

### Modeling of Pol II at the position +49 in a nucleosome and analysis of the structural features of the modeled complex

The model was built manually using mainly the USCF Chimera program (http://www.cgl.ucsf.edu/chimera/) (21) by combining the structure of the nucleosome core particle based on 601 nucleosome positioning sequence (NPS) [PDB ID 3LZ0 (22)] with the structure of yeast Pol II elongation complex [PDB ID 1Y1W (23)].The 3D-DART web server was used to regenerate DNA conformation (24). Other criteria followed the description in published paper (20). A *T.th.* EC+49 was built by superimposing the RNAP crystal structure [PDB ID: 2O5I (25)] to Pol II EC+49 model. The molecular electrostatic surfaces of the proteins were calculated using APBS (26) and displayed by PyMOL (http://www.pymol.org). The distances between the Pol II negatively charged surface and the histone octamer and the residues of amino acids on the surface have also been identified by PyMOL. All protein sequences of the RNAPs in this article were referred from the NCBI reference sequence database (NP_000928.1, NP_010141.1, NP_418415.1, YP_145078.1 and CAB65466.3) (http://www.ncbi.nlm.nih.gov/ guide/) (Supplementary Table S2).The sequences of various RNAPs were aligned by NCBI Blast (http://blast.ncbi.nlm.nih.gov) by the composition matrix adjustment method.

### Cloning *Thermus thermophilus rpo* genes in *Escherichia coli,* expression and co-expression plasmids

*Thermus thermophilus* (HB8 strain) genomic DNA (Refseq: NC_006461) has been used to PCR-amplify *rpoA, rpoB, rpoC, rpoZ* and *rpoD* genes and to clone them in pET series *E. coli* expression vectors. Plasmids pET28-TthA, pET28-TthB, pET28-TthC, pET28-TthZ and pET28-TthD overexpressing, respectively, untagged *T. thermophilus* RNAP α and β subunits, N-terminally hexahistidine-tagged β′, untagged ω and N-terminally hexahistidine-tagged σ^A^ subunits were constructed using routine cloning methods. Plasmid pET28-TthABCZ co-overexpressing RNAP subunits sufficient for the assembly of catalytically proficient *T. thermophilus* RNAP core enzyme was created in two steps by using the single-*rpo*-plasmids described above. In all the plasmids a T7 RNAP promoter precedes each of the *rpo* genes.

### Protein purification

Yeast Pol II [wild-type (WT) and mutant (MU)] and core histones were purified using published protocols (20). *Thermus thermophilus* and *Thermus aquaticus* RNAPs and their sigma factors were purified as described (27). The -H1 chicken erythrocyte chromatin as the donor of histones for nucleosome assembly was purified as described (28).

### Yeast strain construction

Strain YDC412 (MATalpha ura3-52 trp1 leu2delta1 his3delta200 pep4::HIS3 prb1delta1.6R can1 GAL RPB3-6His::URA3 rpb1-E1404A-E1407A-E1411A::LEU2) has three point mutations in the RPB1 gene: E1404A (GAA to GCA), E1407A (GAA to GCG, creating an NruI site) and E1411A (GAA to GCA, creating a PvuII site). YDC412 was obtained by transformation of BJ5464 Rpb3 His-Bio (29) with a SpeI-XhoI digest of p705. Plasmid p705 contains nucleotides 3836–4446 of RPB1, relative to the start codon, including the 3′-coding region with the mutations and the 3′-UTR of RPB1 with LEU2 inserted at the SwaI site in the same orientation as RPB1. Leu+ colonies were screened by PCR and restriction digestion; the mutations were confirmed by sequencing a PCR fragment derived from genomic DNA.

### DNA template construction and nucleosome reconstitution

Templates: λ-603 and λ-603-49 contained λ promoter (30), a 50-bp linker DNA and the 603 NPS (20). The 603 templates were prepared by annealing pairs of long overlapping oligonucleotides and filling-in with the Klenow fragment of DNA polymerase I as described (20). A L603 templates used in Pol II transcription experiment has been published (20). The complete DNA templates were then amplified by PCR reaction and purified from gel electrophoresis using a gel extraction kit (Omega Bio-Tek). We reconstituted nucleosomes on the DNA templates by histone octamer transfer from chicken -H1 erythrocyte donor chromatin (16).

### Transcription of nucleosomes and DNase I footprinting

The *in vitro* transcription assay with *Thermus’* RNAPs was similar to the published protocol (20). In short, *Thermus’* RNAPs were first activated at 65°C in transcription buffer (TB) (20 mM Tris HCl, pH 8.0, 5 mM MgCl_2_, 2 mM β-mercaptoethanol) for 5 min and then shifted to 37°C. The elongation complexes containing 26-mer RNA (EC-17) on pre-assembled nucleosomal templates as was described (20). In experiments with labeled RNA, the 17-mer was pulse-labeled in the presence of [α-^32^P]-GTP (3000 Ci/mmol, PerkinElmer Life Sciences). EC-17 was extended in the presence of 400 mM NTPs at 25°C in the TBs having different concentrations of KCl (20 mM Tris HCl, pH 8.0, 5 mM MgCl_2_, 2 mM β-mercaptoethanol, 40–300 mM KCl). In footprinting experiments with *T. aquaticus* RNAP, all steps were performed in solution. EC+49 complexes were formed in the presence of 1 µM ATP on the 603-49 templates. EC-5 was assembled first and then further extended in the presence of 300 µM CTP, UTP, GTP and 300 µM 3′dATP at 25°C for 4 min in TB300. Labeled DNA was purified from native PAGE and separated by denaturing PAGE. To analyse the nucleosome fate after transcription, the DNA-labeled templates were transcribed and analysed by native PAGE as described (19). The data were quantified using ImageQuant software. DNase I footprinting was conducted as described (20).

The *in vitro* transcription assay with WT or MU yeast Pol II was performed as described (20).

### Analysis of the fate of nucleosomes after transcription using restriction enzyme sensitivity assay

The *in vitro* transcription with *T. aquaticus* RNAP was conducted using ^32^P-labeled λ-603-49 template in the TB300 buffer (20). EC-39 was formed in the presence of ATP and GTPs, and transcription was continued in the presence of all NTPs. Then the buffer was diluted to 100 mM KCl and the templates were incubated in the presence of an excess of restriction enzyme *Cla I* having a single intranucleosomal site at the position +101, before or after transcription for 30 min at 20°C. End-labeled DNA was purified and analysed by native and denaturing PAGE. The amounts of digested DNA were quantified and plotted as fraction of all templates transcribed to completion (as determined by native PAGE). Averages from three experiments and standard deviations were determined.

## RESULTS

### Modeling the nucleosomal Ø-loop-containing Pol II elongation complex (EC+49)

**Figure 1.**
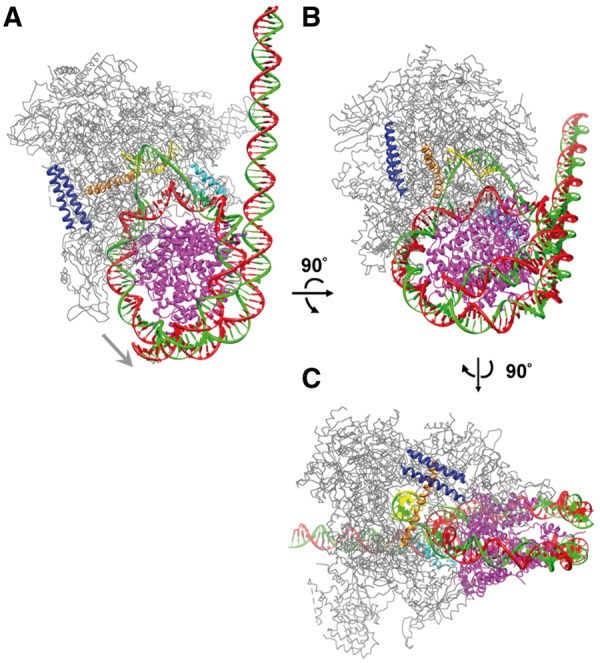
Model of EC+49 (Ø-loop) with yeast Pol II. (**A**) The structures of a nucleosome and a yeast Pol II elongation complex with the active site at the position +49 [PDB 3LZ0 and 1Y1W, respectively (22,23)] were merged using the docking approach. To allow formation of the small intranucleosomal DNA loop containing transcribing Pol II, a ∼50-bp promoter-distal region of nucleosomal DNA has been uncoiled from the octamer (20). The nucleosome octamer is depicted in magenta. The DNA template, non-template and RNA strands are in green, red and yellow, respectively. The bridge helix, the clamp, the C-terminal coiled coil and the rest of the Pol II molecule are shown in orange, cyan, blue and gray, respectively. The gray arrow indicates direction of transcription. (**B**) The structure was rotated by ∼90° around the horizontal axis. (**C**) The structure was further rotated by ∼90° around the vertical axis.

Our previous studies have identified the Ø-loop-containing +49 elongation complex (EC+49, 49 bp from the promoter-proximal nucleosomal boundary) as the key intermediate mediating nucleosome survival and a similar complex (EC+39) has been modeled using docking approach (20). The EC+39 and EC+49 complexes are expected to have similar structures because DNA-binding regions on the surface of the histone octamer are repeated over every 10-bp intervals (31). However it is important to model the authentic EC+49 complex because detailed structures of EC+39 and EC+49 are different. We modeled the EC+49 by docking the high-resolution structures of yeast Pol II EC onto the nucleosome with 601 NPS (22,23) (Figure 1).

The EC+39 and EC+49 share the following properties (Figure 1): (i) The bulk of the Pol II molecule faces into solution and there are no steric clashes with core histones. (ii) The 90^o^ DNA bend present in the EC faces the octamer surface and allows formation of the Ø-loop. (iii) DNA-histone contacts with ∼20-bp DNA region behind the EC stabilize the Ø-loop. (iv) Displacement of ≥50 bp from the promoter-distal end of the nucleosome reduces the size of the DNA region interacting with histones in front of the enzyme from ∼100 to ≤50 bp. This likely facilitates further uncoiling of DNA from the octamer ahead of Pol II and transcription through the nucleosome (20). (v) The negatively charged region on the surface of Pol II (putative Ø-loop-stabilizing sequence, ZLS region) is in close proximity to a positively charged region of the histone octamer and thus could stabilize the Ø-loop and facilitate nucleosome survival during transcription (see below).

### Identification of negatively charged regions on putative histone-interacting Pol II surface

**Figure 2.**
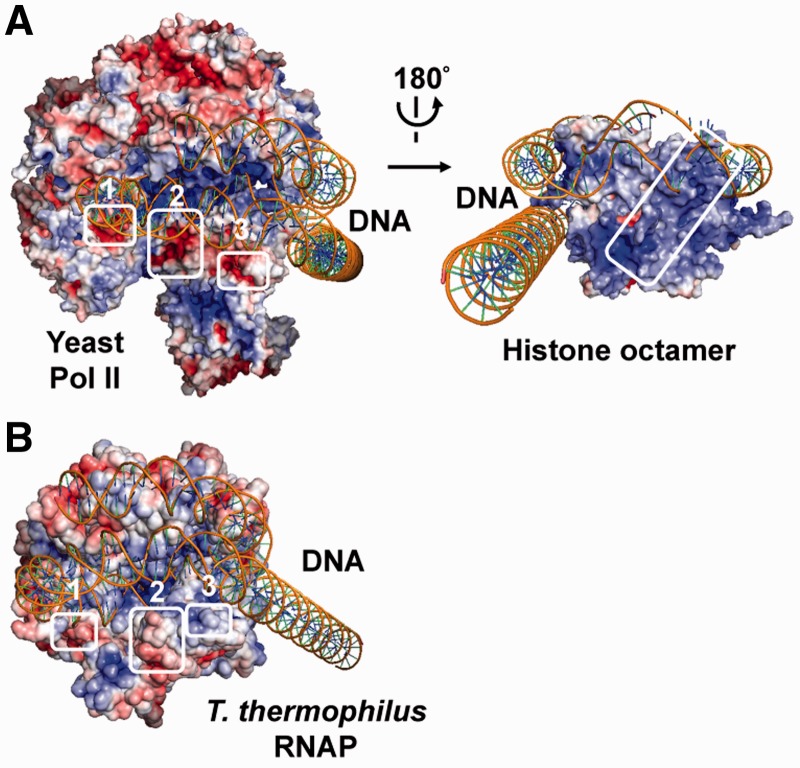
Negatively charged surface of Pol II could stabilize the Ø-loop intermediate through interaction with the histone octamer. (**A**) Putative histone-interacting molecular surfaces of yPol II. On the left: Three identified surfaces on the large subunit Rpb1 of yeast Pol II within the EC+49 (modeled as in Figure 1), interacting with the histone octamer (not shown), are shown by white squares (regions 1–3). On the right: The contacting surface on histone octamer (rotated 180° around the vertical axis) is shown. The darkest blue and the darkest red denote electrostatic potential of 5 kT/e and −5 kT/e, respectively. DNA is shown in yellow. (**B**) Molecular surfaces of *T. thermophilus* RNAP (PDB ID 2O5I) in the hypothetical model of EC+49 complex are colored by the magnitude of the electrostatic potential (histone octamer is not shown). Regions 1–3 are homologous to histone-contacting surfaces of Pol II (Figure 2A).

Using the EC+49 model (Figure 1), putative Pol II-interacting surface on the histone octamer and three histone-interacting surfaces of Pol II were identified (Figure 2A). All histone-interacting surfaces are negatively charged and localized on the largest subunit of the yeast Pol II (yRpb1, regions 1–3, Figure 2A). To further evaluate the possible role of the putative ZLS regions on Pol II during transcription through chromatin, their presence and conservation in various structurally related eukaryotic and prokaryotic RNAPs has been evaluated (Table 1 and Supplementary Table S1). *Escherichia coli* RNAP that utilizes the Pol II-type mechanism of transcription through chromatin (32) was not included in this study because the high-resolution structure of this enzyme is not available. The sequence of ZLS regions 2 and 3 are conserved with more than 50% sequence identity in multi-subunit prokaryotic RNAPs from *T. thermophilus* and *T. aquaticus* that are structurally similar to Pol II (25,33), whereas the region 1 is only conserved in eukaryotic RNAPs. No homologous sequences were identified in the single-subunit bacteriophage T7 RNAP.

**Table 1.** Correlation between the presence of the negatively charged regions on octamer-facing surface of RNAPs, the height of the nucleosomal barrier and nucleosome survival during transcription

Enzyme	Species	ZLS regions net charge^a^	Ø-loop at +49	Height of +45 barrier	Nucleosome survival
Pol II (RPB1)	*H. sapiens*	−8	ND	+++++^b^	ND
Pol II (Rpb1)	*S. cerevisiae*	−15	+^c^	+++++^b^	+++++^d^
RNAP (*β*′)	*T. thermophilus*	+1	ND	+^e^	ND
RNAP (*β*′)	*T. aquaticus*	+1	−^e^	+^e^	–

^a^The net charge of the region homologous to the ZLS region 1434–1450 of Rpb1 subunit of yeast Pol II.

^b^Reference (34).

^c^Reference (20).

^d^Reference (32).

^e^This work.

It has been proposed that the net negative charge of the ZLS sequence could be important for stabilization of the Ø-loop and efficient nucleosome survival during Pol II transcription (20). In agreement with this proposal, the ZLS regions are negatively charged in yeast Pol II (Figure 2A) that does not displace nucleosomes during transcription (16,32). The ZLS regions of human Pol II are also negatively charged and thus human Pol II is expected to preserve nucleosomal structure during transcription. This proposal is consistent with the observed similar patterns of strong nucleosomal pausing characteristic for human and yeast Pol II (34).

In contrast, the negative charges of the ZLS regions are not preserved in other analysed prokaryotic RNAPs (from *T. thermophilus* and *T. aquaticus*) (Figure 2B and Table 1). Although the putative histone-interacting surface of *T. thermophilus* RNAP has some regions having local negative charge (Figure 2B), there is no net negative charge within the region (Table 1). The data suggest that the net negative charge within the histone-interacting surface of these RNAPs positively correlates with the probability of nucleosome survival during transcription. Furthermore, the distance between ZLS regions and the histone octamer surface is on average ∼5 Å larger for *T. thermophilus* RNAP than for yeast Pol II. The lower net negative charge of the ZLS regions and the larger distance between RNAP and the histone octamer likely decrease the strength of RNAP–histone interactions. Accordingly, RNAPs having lower net negative charge of the ZLS region are expected to form less stable Ø-loop-containing EC+49. Since stable Ø-loop is likely essential for nucleosome survival, the efficiency of survival is expected to be lower during transcription by *T. thermophilus* and *T. aquaticus* RNAPs, as compared with Pol II. Because stable Ø-loop dictates strong nucleosomal pausing at the +45 region (35), a decrease in the +45 pausing is also expected during transcription by these RNAPs. To evaluate these predictions of the model, identical DNA and nucleosomal templates were transcribed by *T. thermophilus* and *T. aquaticus* RNAPs.

### *Thermus thermophilus* and *T. aquaticus* RNAPs efficiently overcome the nucleosomal barrier to transcription

**Figure 3.**
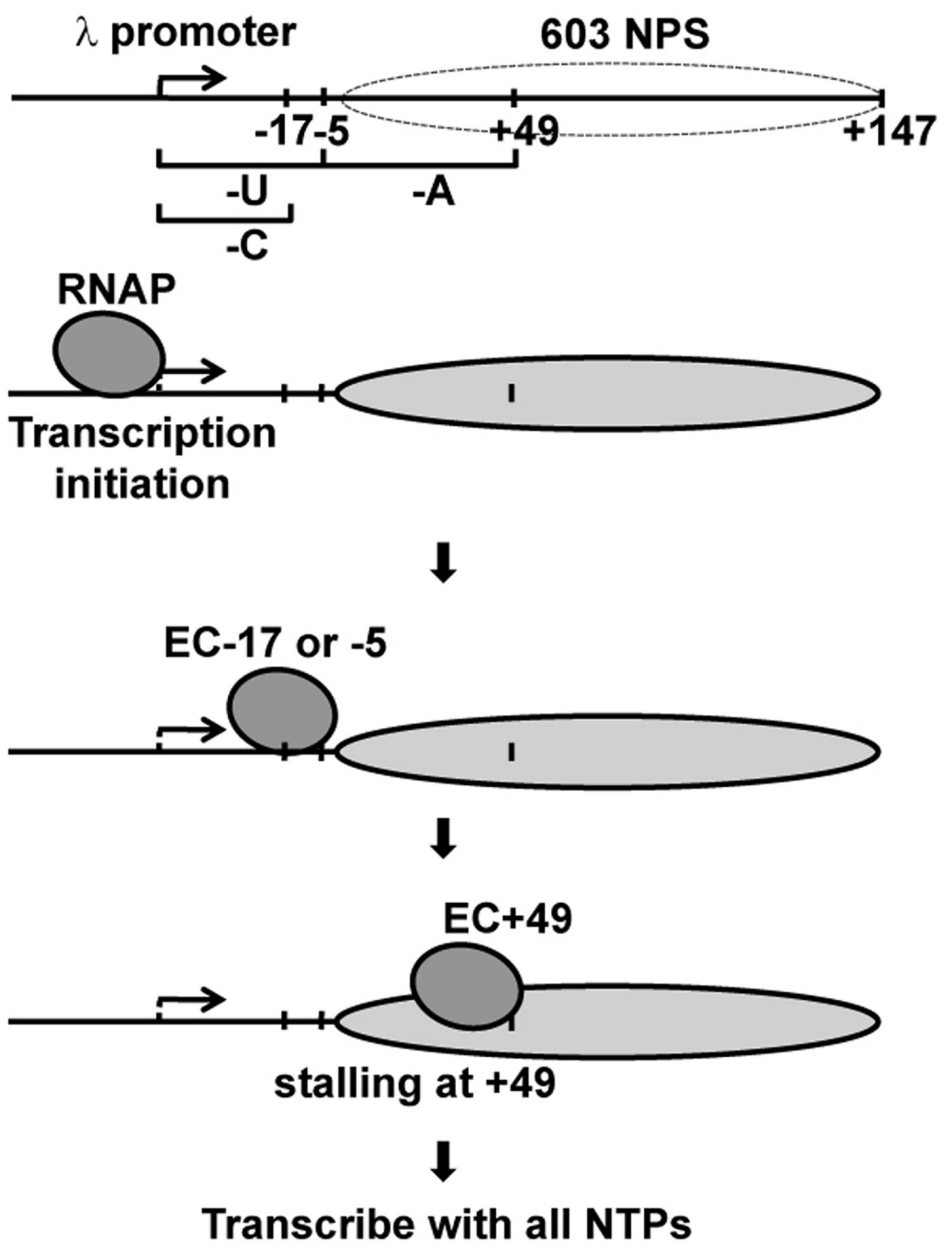
Experimental strategy for stalling the transcript elongation complexes at unique positions on the nucleosomal templates. Each template contains the λ promoter and allows progression to and stalling of the ECs at the –5, –17 and +49 positions (relative to the boundary of nucleosome formed at the strong 603 NPS) upon addition of different partial combinations of NTPs. After transcription initiation, RNAP was stalled at the position –5 or –17. Next, RNAP was stalled at the position +49 within the nucleosomes in the presence of a subset of NTPs or transcribed in the presence of all NTPs.

To construct defined DNA and mononucleosomal templates transcribed by *T. thermophilus* and *T. aquaticus* RNAPs *in vitro*, the λ promoter has been placed upstream of the strong 603 NPSs (603 NPS, Figure 3). The λ promoter maintains initiation by both RNAPs. The 603 NPS is a NPS with high affinity to histone octamer that maintains unique nucleosome positioning and allows structural analysis of transcribed nucleosomes (20,36). On the 603 template, the RNAP can be stalled at specific positions (Figure 3): before entering the nucleosome (the –17 or –5 positions on different sequence versions of the template) and at the position +49.

**Figure 4.**
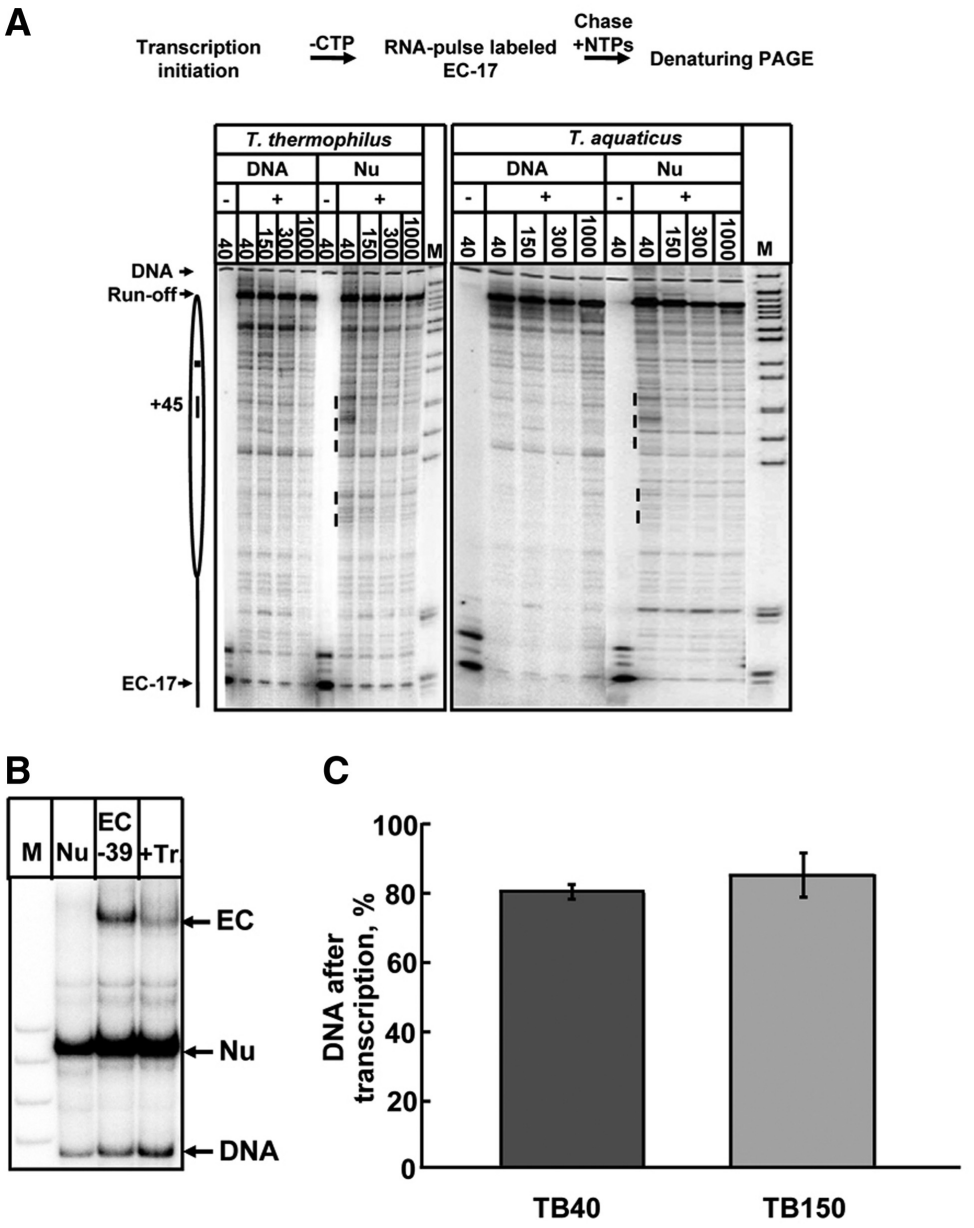
Analysis of transcription through a nucleosome by *T. thermophilus* and *T. aquaticus* RNAPs. (**A**) The experimental approach for analysis of the nucleosomal barriers to transcription by *T. thermophilus* and *T. aquaticus* RNAPs is shown at the top. Positioned 603 nucleosomes were assembled downstream of the transcription initiation sites. Transcription was initiated in the presence of α-P^32^-GTP and various RNAPs were stalled at the position –17 relative to the boundary of the nucleosome. Then transcription was continued at the indicated KCl concentrations in the presence of all unlabeled NTPs and rifampicin that limits transcription to a single round. Pulse-labeled RNA was analysed by denaturing PAGE. The locations of the nucleosome (oval), nucleosome dyad (square), EC-17 and the run-off transcript are shown. Dashed line: nucleosome-specific pausing. Note that even at 40 and 150 mM KCl the 603 nucleosome presents a low barrier for the RNAPs. M indicates *Msp*I digest of pBR322 plasmid. (**B**) The 603 nucleosome is disrupted after transcription by *T. aquaticus* RNAP: analysis in native PAGE. Transcription (+Tr.) was conducted at 300 mM KCl after pre-formation of the EC-39. DNA-labeled templates were analysed by native PAGE. The amounts of histone-free DNA produced after transcription were quantified (Figure 4C). Note that nearly all active elongation complexes are converted into DNA. (**C**) Quantitative analysis of the data (Figure 4B).The amounts of histone-free DNA produced after transcription were quantified and plotted as fraction of all templates transcribed to completion (active elongation complexes converted into the products of transcription, Figure 4B). Averages from three experiments and standard deviations are shown. Note that the majority of nucleosomes (∼80%) are converted into histone-free DNA after transcription.

Transcription through the 603 nucleosome by yeast Pol II is accompanied by a strong nucleosome-specific pausing observed at 40, 150 and 300 mM KCl and the run-off transcripts are produced only on a small fraction (5–25%) of nucleosomal templates at 40 and 150 mM KCl (34). To analyse the efficiencies of transcription through the 603 nucleosome by *T. thermophilus* and *T. aquaticus* RNAPs, limited transcription was conducted in the presence of labeled GTP, RNAPs were stalled at the position –17 and then chased in the presence of an excess of all unlabeled NTPs at different salt concentrations (40, 150, 300 and 1000 mM KCl). The pulse-labeled RNA was analysed by denaturing PAGE (Figure 4A).

In contrast to strong nucleosomal pausing characteristic for Pol II (34), nucleosome-specific pausing during transcription by *T. thermophilus* and *T. aquaticus* RNAPs was much less pronounced and the yield of the run-off transcripts was more than 80%. In particular, only very limited nucleosome-specific pausing at the +45 region was observed during transcription by *T. thermophilus* and *T. aquaticus* RNAPs (especially at 150 and 300 mM KCl, Figure 4A), indicating that most likely formation of the Ø-loop occurs with lower efficiency than during transcription by Pol II.

### The nucleosome is displaced by transcribing *T. aquaticus* RNAP

To evaluate the fate of the nucleosome on transcription by *T. aquaticus* RNAP, the 603 nucleosomal templates were analysed in a native gel before and after transcription. Nucleosomes were formed at the desired position on the templates with more than 95% efficiency (Supplementary Figure S1).

Transcription by yeast Pol II results in displacement of the histone octamer from ∼20% of templates and conversion of remaining nucleosomes into the hexasomes remaining at the original position on the template (19). In contrast, more than 80% of transcribed templates are converted into histone-free DNA after transcription by *T. aquaticus* RNAP, as was determined by analysis by native PAGE (Figure 4B and C) and analysis of sensitivity of nucleosomes before and after transcription to restriction enzyme *Cla I* having a single intranucleosomal site at the position +101. The data on analysis of nucleosomal pausing (Figure 4A) and the fate of nucleosomes on transcription by *T. aquaticus* RNAP are consistent and suggest that more efficient transcription through the nucleosome by *T. aquaticus* RNAP is likely explained by less efficient formation of the Ø-loop-containing intermediate (EC+49) and nucleosome displacement during transcription by this enzyme (see Supplementary Discussion).

### *Thermus aquaticus* RNAP does not form the Ø-loop at the position +49

**Figure 5.**
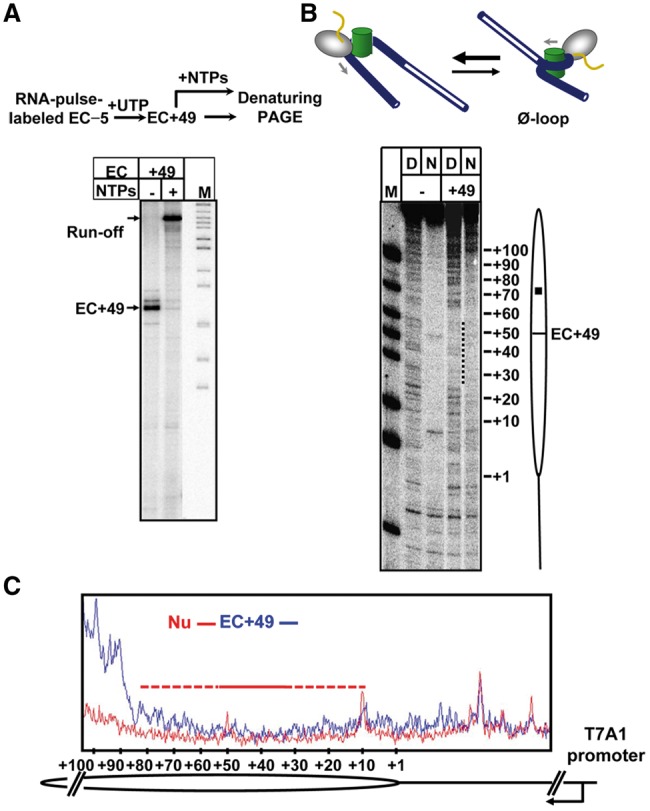
EC+49 formed by *T. aquaticus* RNAP does not contain the Ø-loop. (**A**) Analysis of the homogeneity and stability of the EC+49. Top: The experimental approach. Bottom: The EC+49 was formed on the 603 nucleosome and then extended in the presence of all NTPs. Analysis of pulse-labeled RNA by denaturing PAGE. Note that the EC+49 is functional and contains extendable RNA. (**B**) The structures of the EC+49 formed on histone-free DNA (D) or nucleosomal templates (N) were analysed by DNase I footprinting. The footprints of the EC+49 (dotted line) and the position of the active center of the RNAP are indicated. Analysis of end-labeled DNA by denaturing PAGE. The suggested structure of the EC+49 complex formed on the 603 nucleosome is shown on the top. (**C**) Quantitative analysis of DNase I footprinting data. Lanes 3 (Nu) and 4 (EC+49 in the 603 nucleosome) in Figure 5B were scanned and the scans were aligned pairwise. Note that formation of the EC+49 results in full DNA uncoiling at the +(1–10) and +(80–150) regions and partial uncoiling at +(10–30) and +(50–80) regions (red-dashed line). The (+30–50) region is strongly protected by RNAP (red solid line). The positions of the nucleosome (oval), nucleosomal dyad (square) and the T7A1 promoter are indicated.

To directly evaluate the efficiency of formation and the structure of the EC+49 during transcription through the nucleosome by *T. aquaticus* RNAP, the enzyme has been stalled at the position +49 (Figure 3). The labeled RNA product was more than 80% homogenous; the EC+49 was stable and fully capable to restart transcription and produce the run-off RNA upon addition of all NTPs (Figure 5A), suggesting that the majority of stalled EC+49 complexes remain transcriptionally active.

Previously, it has been shown that yeast Pol II and *E. coli* RNAP stalled at the position +49 within the 603 nucleosome form a Ø-loop-containing complex where DNA is tightly wrapped around the octamer both in front and behind of the transcribing enzyme and is tightly protected from digestion by DNase I and restriction enzymes (20). The structure of EC+49 formed by *T. aquaticus* RNAP on the 603 nucleosome was analysed using DNase I footprinting. The complex was partially digested by endonuclease DNase I and end-labeled DNA was analysed by denaturing PAGE (Figure 5B and C and Supplementary Figure S2). DNA both in front and behind of the enzyme is much more accessible to DNase I digestion in nucleosomal EC+49 than in the original nucleosome. Furthermore, the pattern of DNA digestion by DNase I downstream of the enzyme in the EC+49 is very similar with the pattern characteristic for histone-free DNA, indicating that in the majority of the complexes DNA downstream of the enzyme is displaced from the octamer. At the same time, ∼15-bp DNA region upstream of nucleosomal EC+49 is partially protected from DNase I, indicating binding of histones to a fraction of EC+49 complexes. Thus in the EC+49 complex formed by *T. aquaticus* RNAP all downstream and the majority of upstream DNA–histone interactions are disrupted. Core histones are at least partially displaced from the nucleosome by the enzyme and the canonical Ø-loop is not formed. Such disrupted DNA–histone interactions are not expected to cause strong pausing; further transcription may result in displacement of the entire histone octamer, as was indeed observed experimentally (Figure 4B and C).

### Negative charge on the surface of Pol II affects transcription through the nucleosome

**Figure 6.**
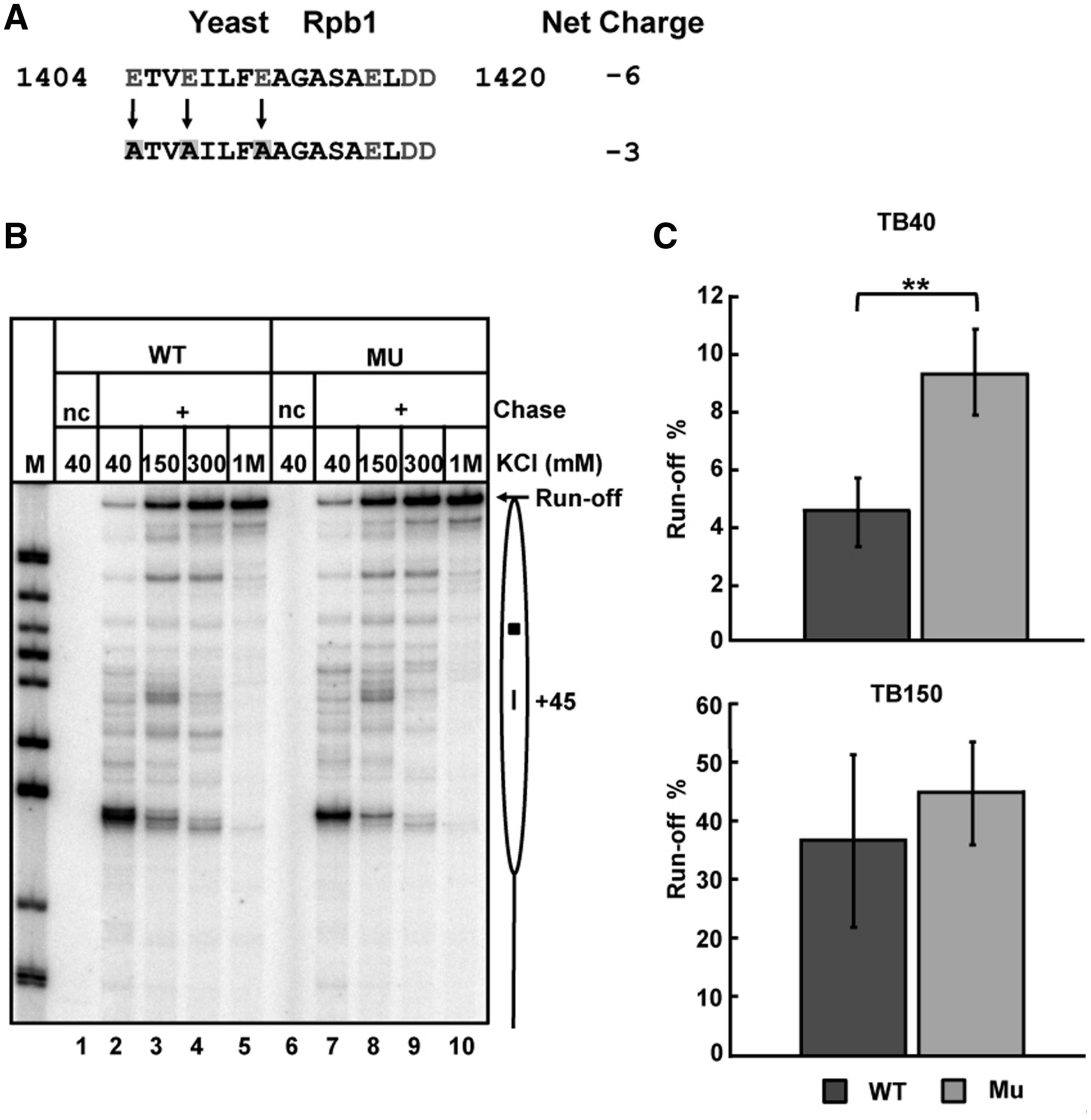
MU Pol II having a lower negative charge of the histone-interacting surface encounters a lower nucleosomal barrier. (**A**) Strategy of Pol II mutagenesis. Three Glu amino acids of yRpb1 subunit at the positions 1404, 1407 and 1411 were simultaneously converted to Ala (marked by yellow squares), changing the net charge of the ZLS region 2 from –6 to –3. Negatively charged amino acids are shown in bold red. (**B**) Transcription of 603 nucleosomal templates by WT and MU yPol II at the indicated KCl concentrations. nc indicates no chase. Pulse-labeled RNA was analysed by denaturing PAGE. Other designations are as in Figure 4. (**C**) Quantitation of run-off transcripts produced at 40 mM KCl and 150 mM KCl by WT or Mu yPol II (Figure 7B). Averages from three experiments and standard deviations are shown (** indicates *P*-value < 0.01).

Finally, the effect of neutralizing negative charge on the putative ZLS Pol II surface has been evaluated. To construct an MU Pol II having lower negative charge on the ZLS surface, three negative charged glutamate residues within the region 2 (yRpb1: 1404E, 1407E, 1411E) were substituted with non-polar alanines (Figure 6A). WT and the MU yPol II complexes were purified. Since the intensity of +45 pausing and efficient formation of the Ø-loop positively correlate with nucleosome survival (35), we expected that the MU Pol II having lower negative charge of the region 2 would encounter a lower nucleosomal +45 barrier. To evaluate this prediction, the nucleosome was transcribed using WT or MU Pol II at 40, 150, 300 or 1000 mM KCl. Pulse-labeled transcripts were analysed by denaturing gel (Figure 6B). As expected, the yield (fraction) of run-off transcripts was higher when transcription was conducted by the MU Pol II, especially at 40 mM KCl (Figure 6B and C and Supplementary Figure S3). The WT and the MU yPol II have similar processivities and transcribe DNA at similar rates (Supplementary Figure S4), suggesting that these properties are not strongly affected by the mutations. The data suggest that higher negative charge of the ZLS Pol II region positively correlates with stronger +45 pausing, likely because it more efficiently interacts with the histone octamer and therefore facilitates efficient formation of the Ø-loop.

### Higher negative charge of ZLS region correlates with a stronger +45 barrier and more efficient nucleosome survival during transcription

The data on the net charge of ZLS region, the height of +45 nucleosomal pausing and the nucleosome fate on transcription by different RNAPs are summarized in Table 1. The presence of a higher negative charge of the ZLS region positively correlates with formation of the key intermediate that strongly facilitates nucleosome survival (Ø-loop at the position +49), with a stronger nucleosomal pausing at the +45 region, and with more efficient nucleosome survival during transcription.

Overall, the data suggest that the presence and net charge of the ZLS region in part determine the structure of the key intermediate formed at the position +49, and, in turn, the nucleosome fate on transcription. When the ZLS region is present and negatively charged (e.g. human and yeast Pol II), nucleosomal +45 pausing is strong, Ø-loop forms at the position +49 and nucleosomes efficiently survive during transcription. When the ZLS region is present, but does not contain negative charge (e.g. *T. thermophilus* and *T. aquaticus* RNAPs), nucleosomal +45 pausing is weak, the Ø-loop does not form, and nucleosomes are lost during transcription.

## DISCUSSION

In our work, the role of the negatively charged region on the surface of Pol II (ZLS regions) during transcription through chromatin has been evaluated using computational modeling and experimental analysis of the mechanism of transcription through chromatin by various RNAPs. Computational modeling suggested that in the elongation complex that determines nucleosome survival during transcription (EC+49) three ZLS regions are in close proximity to a positively charged region of the histone octamer and thus could stabilize the Ø-loop and facilitate nucleosome survival during transcription (Figures 1 and 2). Analysis of transcription by RNAPs from *T. thermophilus* and *T. aquaticus* that have uncharged ZLS regions suggests that in this case the nucleosomal +45 pausing is weak, the Ø-loop does not form, and nucleosomes are lost during transcription (Figures 4 and 5). In contrast, transcription by Pol II having negatively charged ZLS regions results in strong nucleosomal +45 pausing, formation of the Ø-loop and nucleosome survival (16,20,34). The strength of the +45 pausing is determined in part by the negative charge present on the ZLS region of Pol II (Figure 6). Thus the properties (in particular, the charge) of the ZLS region dictate all key parameters of the mechanism of transcription through the nucleosome.

How can the presence and charge of the ZLS region affect these multiple aspects of transcription through chromatin? Previously we have proposed that the ZLS region could affect the structure/stability of the key Ø-loop-containing intermediate (EC+49) formed during transcription through a nucleosome (20). This intermediate, in turn, was expected to couple the strong nucleosomal pausing at the +45 region with efficient nucleosome survival during transcription (35). In agreement with this proposal, our current data suggest that RNAPs missing or having an uncharged ZLS region do not encounter the strong nucleosome barrier, do not form the Ø-loop and do not maintain efficient nucleosome survival.

**Figure 7.**
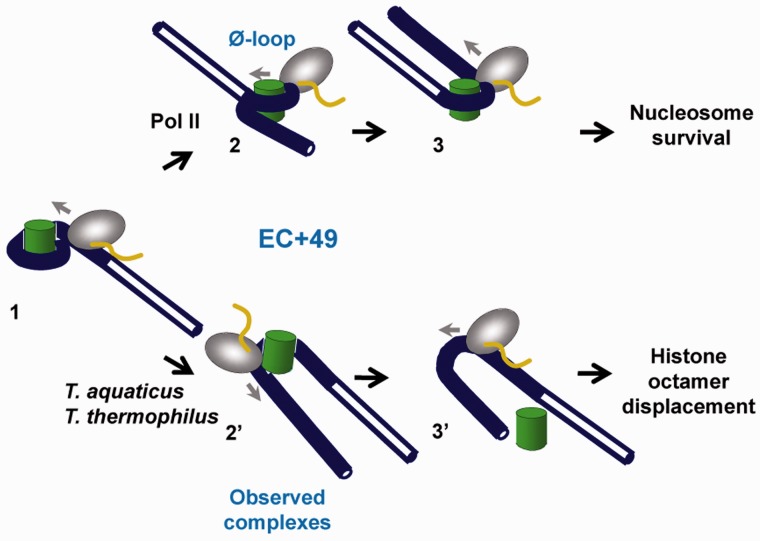
Proposed mechanisms of transcription through a nucleosome by different RNAPs. We propose that the overall negative charge on the surface of RNAP that faces the histone octamer in part dictates the structure of the critical intermediate complex (EC+49). The structure of the EC+49, in turn, determines the fate of the nucleosome on transcription. As different RNAPs enter the nucleosome (complex 1), they form structurally different complexes at the position +49. Specifically, when a higher negative charge is present on the surface of RNAP (human and yeast Pol IIs, Table 1), a stable, Ø-loop-containing intermediate 2 is formed. Formation of such a complex results in uncoiling of the downstream nucleosomal DNA (3) and nucleosome survival (20). Alternatively, when the surface of RNAP contains a lower negative charge (*T. thermophilus* and *T. aquaticus* RNAPs), an unstable EC+49 is formed (complex 2′); the histone octamer is loosely bound to the EC and is lost during further transcription (3′).

Based on our data, we propose the following model explaining the observed relationship between the +45 pausing, Ø-loop formation and nucleosome fate (Figure 7). As different RNAPs enter the nucleosome and approach the +45 region (complex 1), they form the Ø-loop with different efficiencies. In particular, yeast Pol II forms the Ø-loop (2) with high efficiency (20), in part because its ZLS region is negatively charged and stabilizes the Ø-loop by interacting with the positively charged surface of the histone octamer. Formation of the Ø-loop induces a slow step—uncoiling of DNA in front of the enzyme (3)—that is accompanied by extensive nucleosomal pausing. DNA behind RNAP is re-wrapped around the histone octamer and the nucleosome is recovered at the original position of the template (20). In contrast, during transcription by *T. thermophilus* and *T. aquaticus* RNAPs having uncharged ZLS region the Ø-loop (if formed) is unstable (complex 2′). Therefore transcription is accompanied by only minor nucleosomal pausing and by displacement of the histone octamer during further transcription through the nucleosome (3′). Thus the structures of the key intermediates formed during transcription through the +45 region of nucleosomal DNA likely dictate multiple aspects of transcription through chromatin, including nucleosomal pausing and the fate of histones on transcription. The presence and charge of the ZLS region are unlikely to be the only factors that affect the outcome of transcription through chromatin. Thus, the distance between ZLS regions and the histone octamer in the EC+49 is likely important for the stability of the Ø-loop.

The negatively charged ZLS region 2 of yeast Pol II is localized within the switch 2 domain and is likely important for separation of the DNA strands during transcription (23). It is also localized within an acidic domain that affects transcription activation, yeast Pol II activity and is important for normal cell growth (37). Our studies suggest an additional essential function of the ZLS region 2—determining the rate of RNAP progression and the fate of nucleosomes during transcription through chromatin. This function is particularly important for eukaryotic Pol II: in this case the conserved, negatively charged ZLS region most likely facilitates nucleosome survival and maintenance of histone marks during transcription.

## SUPPLEMENTARY DATA

Supplementary Data are available at NAR Online, including [38–40].

## FUNDING

National Institute of Allergy and Infectious Diseases (NIAID) [R21-AI090558 to K.K.]; National Institutes of Health (NIH) [GM58650 to V.M.S.]; Russian Foundation for Basic Research (RFBR) [12-04-31942 to A.K.S.]; Intramural Research Program of the National Institutes of Health (NICHD) (to D.J.C.). Funding for open access charge: Grant of NIH [GM58650 to V.M.S.].

*Conflict of interest statement*. None declared.
